# LRRC3B在非小细胞肺癌中下调并与肺癌细胞增殖和侵袭能力相关

**DOI:** 10.3779/j.issn.1009-3419.2016.04.01

**Published:** 2016-04-20

**Authors:** 亮 阚, 萌 张, 平 何

**Affiliations:** 110004 沈阳，中国医科大学附属盛京医院老年病科 Department of Geriatrics, Shengjing Hospital of China Medical University, Shenyang 110004, China

**Keywords:** 肺肿瘤, LRRC3B, 增殖, 侵袭, 细胞周期, Lung neoplasms, LRRC3B, Proliferation, Invasion, Cell cycle

## Abstract

**背景与目的:**

已有的研究表明：在许多恶性肿瘤细胞中，LRRC3B表达显著下调，被视为肿瘤抑制蛋白。然而，在非小细胞肺癌中它的表达模式和生物学作用缺乏研究。人类癌症微阵列的研究显示LRRC3B在乳腺癌和结肠直肠癌表达下调，提示LRRC3B参与致癌作用。本研究的目的是研究LRRC3B在非小细胞肺癌中的必到状态及其与肺癌增殖、侵袭和细胞周期间的相关性，探讨LRRC3B在调控肺癌细胞增殖、侵袭及细胞周期中的作用。

**方法:**

应用Western blot和Realtime RT-PCR检测LRRC3B在几株肺癌细胞系中的mRNA和蛋白表达水平。应用MTT法检测对转染LRRC3B的A549和H460细胞系细胞增殖能力变化，应用集落形成实验以及细胞侵袭实验研究LRRC3B对细胞增殖和侵袭以及细胞周期进程的作用。肺癌细胞系H3255中转染LRRC3B siRAN验证LRRC3B对细胞的增殖以及侵袭能力和对细胞周期进程的影响。

**结果:**

与正常NHBE细胞系相比，NSCLC细胞系中LRRC3B蛋白表达量显著下调，特别是H460、H358、HCC827以及A549。A549和H460细胞系转染LRRC3B后，细胞增殖和侵袭能力受到抑制。LRRC3B抑制细胞周期进程，并下调cyclin D1和MMP9的表达。H3255细胞中敲除LRRC3B，细胞增殖和侵袭能力显著增强，同时与细胞周期及侵袭能力相关的蛋白cyclin D1和MMP9表达略微上调。

**结论:**

LRRC3B在肺癌细胞系中表达下调，而上调LRRC3B则能够抑制肺癌细胞增殖和侵袭能力，并抑制细胞周期进程，可能是未来肺癌治疗的一个新靶点。

## Abstract (en)

**Background and objective:**

LRRC3B expression is downregulated in many kinds of malignant tumors and it is regarded as tumor inhibition protein. However, the expression pattern and biological effect are still unclear in nonsmall cell lung cancer (NSCLC). Study of human cancer microarray showed LRRC3B was downregulated in breast cancer and colorectal cancer, which declares that LRRC3B takes part in tumor formation. This study is to investigate the expression of LRRC3B in lung cancer cell lines and the influence of LRRC3B during cell proliferation, invasion and cell cycle.

**Methods:**

The expression of LRRC3B was detected through Western blot and Realtime RT-PCR. MTT assay, colony formation assay and matrigel invasion assay were performed to explore the effect of LRRC3B on cell proliferation, invasion and cell cycle. LRRC3B siRAN was transfected in lung cancer cell line H3255, and then the effect of LRRC3B on cell proliferation, invasion and cell cycle was analyzed.

**Results:**

During 4 of 9 lung cancer cell lines, the expression of LRRC3B is downregulated compared with normal epithelial cells. Cell proliferation and invasion were inhibited in A549 and H460 after LRRC3B transfected. LRRC3B suppressed the progress of cell cycle and downregulated the expression of cyclin D1 and MMP9. The activity of proliferation and invasion was promoted after LRRC3B was knocked out in H3255.

**Conclusion:**

LRRC3B expression downreguated in lung cancer cell lines and LRRC3B could inhibit lung cancer cell proliferation, invasion and cell cycle progress. LRRC3B could be a new target for lung cancer therapy.

近年来肺癌发病率持续升高，已跃然成为恶性肿瘤死因的第一位^[1-3]^。尽管对于表皮生长因子受体（epidermal growth factor receptor, *EGFR*）、鼠肉瘤病毒（Kirsten rat sarcoma viral oncogene homolog, *K-RAS*）和间变性淋巴瘤激酶（anaplasticlymphoma kinase, *ALK*）等基因突变的病例可选用靶向药物进行治疗，然而这些基因的突变仅存在于肺腺癌当中的部分病例中。另一方面，各种遗传、表观和微环境因素的相互复杂作用也显著影响肿瘤细胞的生存和侵袭^[4, 5]^。因此，揭示肺癌细胞增殖、侵袭转移的分子机制，发现肺癌侵袭、转移的关键分子或靶点，对于开发治疗肺癌的新的靶向药物具有重要的理论和临床意义。

富含亮氨酸重复序列（leucine rich repeat-containing, LRR-containing）蛋白是具有保守亮氨酸序列的跨膜蛋白。该蛋白参与许多重要的生命进程，包括动植物免疫、细胞粘附、激素受体作用、信号转导、基因表达调控以及凋亡等过程^[6, 7]^。几项人类癌症微阵列的研究显示LRRC3B在乳腺癌和结肠直肠癌表达下调，表明LRRC3B参与致癌作用^[8]^。据报道，LRRC3B mRNA在胃癌组织中表达下调，在裸鼠胃癌细胞中引入LRRC3B则会抑制集落的形成以及肿瘤的发生^[9]^。但是目前还没有报导人类非小细胞肺癌（non-small cell lung cancer, NSCLC）中LRRC3B相关蛋白的表达模式以及临床作用。其在肺癌细胞中的生物学功能也仍然是未知的。我们猜想它可能在肺癌细胞中也是低表达，并抑制一些细胞功能的发挥。我们通过Western blot和Realtime RT-PCR检测了LRRC3B在NSCLC中的表达情况，并采用转染的方法增加LRRC3B的表达，后通过集落形成实验，MTT方法以及侵袭实验验证其对细胞增殖、细胞侵袭的作用以及其可能机制。我们发现，在肺癌细胞中LRRC3B表达下调，并且它能够抑制癌症细胞的增殖和侵袭。现将结果报道如下。

## 1 材料和方法

### 1.1 细胞培养和转染

本课题研究所用人类NSCLC细胞系H460和A549以及H3255购于美国模式培养物保藏中心（American Type Culture Collection, ATCC）。本课题研究所用肺癌细胞系用含有10%热灭活新鲜胎牛血清（Fetal Bovine Serum, FBS）的RPMI-1640（Gibco, Invitrogen, NY, USA）在5%CO_2_，37 ℃培养箱中培养。

### 1.2 质粒和siRNA转染

pCMV6-LRRC3B质粒和对照空pCMV6从Origene（Origene, Rockville, MD, USA）购买。采用attractene转染试剂（Qiagen, Hilden, Germany）进行转染。siLRRC3B序列和对照siRNA序列从Dharmacon（ThermoFisher, USA）购买。采用DharmaFECT 1试剂进行干扰。

### 1.3 Real-time RT-PCR

Real-time PCR采用ABI（Applied Biosystems）SYBR Green mastermix试剂盒。使用ABI 7500快速实时定量PCR系统，目的基因的相对表达量通过2^-ΔΔCt^方法计算。引物序列如下：LRRC3B Forward：5'TCCAATCATGAGACAGCCCAC 3'，LRRC3B Reverse：5'TCTGCCAGCATGTTCATCCAA 3'；β-actin Forward：5' ATAGCACAGCCTGGATAGCAACGTAC 3'，β-actin Reverse：5' CACCTTCTACAATGAGCTGCGTGTG 3'。

### 1.4 Western blot

总蛋白使用Pierce裂解液（ThermoFisher, USA）提取。Bradford方法进行蛋白定量。上样蛋白量为60 μg。电泳、转印（50 V, 120 min）、5%脱脂奶粉封闭，抗LRRC3B（1:800, Sigma, USA）抗cyclinD1、MMP9（1:1, 000, Cell Signaling, USA），4 ℃孵育过夜，分别与对应的二抗（1:2, 500, Santa Cruz, USA）37 ℃孵育2 h，ECL显色，结果经自动电泳凝胶成像分析仪（DNR, Jerusalem, Israe）采集^[10]^。

### 1.5 MTT法检测细胞增殖

转染后将单个细胞悬液接种于96孔培养板中，每孔约2, 000个细胞，培养24 h，每孔加入20 mL 3-（4, 5-二甲基噻唑-2）-2, 5-二苯基四氮唑溴盐（3-[4, 5-2-yl]-2, 5-diphenyltetrazolium bromide, MTT）溶液（5 mg/mL, Sigma, USA）继续培养4 h，吸去上清液，加入150 μL二甲基亚砜（DMSO, Sigma, USA），振荡10 min，490 nm波长下测定各孔光吸收值，实验重复3次，以不含细胞的等体积培养基作对照。绘制细胞生长曲线^[11]^。

### 1.6 集落形成实验

集落形成实验在转染48 h后接种1, 000个细胞在6 cm培养皿中2周后用吉姆萨染色液（Giemsa）染色。以50个细胞计为是1个克隆。

### 1.7 细胞侵袭实验

细胞侵袭实验使用有8 μm孔径膜的24-well Transwell（Costar, Cambridge, MA）膜被20 μL胶（1:3, BD Bioscience, San Jose, CA, USA）覆盖。转染48 h后，胰蛋白酶消化细胞，含3×10^5^个细胞的100 μL无血清培养基转移到人工基底膜室上部并孵化16 h。中途补充10%FBS到下室作为引诱物。培养后，上室表面未侵袭的细胞用棉花擦除，通过孔径的细胞用4%多聚甲醛固定并用苏木精染色。在显微镜下随机选择10个视野统计入侵细胞，实验重复3次。

### 1.8 统计学方法

采用SPSS 16.0软件进行数据分析，实验结果采用*t*检验进行分析，数据采用Mean±SD表示，以*P* < 0.05为差异有统计学意义。

## 2 结果

### 2.1 在肺癌细胞系中LRRC3B下调并抑制细胞增殖和侵袭

肺癌细胞系中通过Western blot和Real time RT-PCR分析LRRC3B的相对表达水平。与组织样本一致，与正常NHBE细胞系相比，NSCLC细胞系中LRRC3B蛋白表达量显著下调，特别是H460、H358、HCC827以及A549（图 1A）。选用A549和H460细胞系进行LRRC3B质粒转染来上调LRRC3B水平。通过Western blot验证转染效率（图 1B）。

**1:**
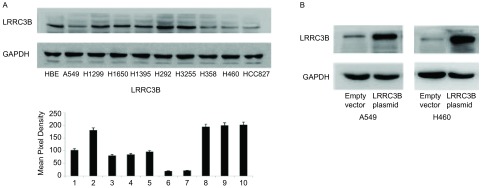
LRRC3B在肺癌细胞系中的表达以及转染效率。A：通过Western blot以及Real-time RT-PCR检测NHBE和几株肺癌细胞系中LRRC3B的表达，与NHBE相比，A549，H358，H460和HCC827中LRRC3B的表达明显下调。B：Western blot分析显示：与对照组相比，在A549和H460细胞中，LRRC3B转染显著增加了LRRC3B的水平。 The expression of LRRC3B in lung cancer cells and transfection efficiency of LRRC3B in A549 & H460. A: The expression of LRRC3B is detected in NHBE and several lung cancer cells through western blot and Real-time RT-PCR. The expression of LRRC3B is downregulated in A549, H358, H460 and HCC827 cells; B: The analysis result indicates that LRRC3B transfection upregulates the expression of protein LRRC3B in A549 and H460 cells.

在A549和H460细胞系中上调LRRC3B水平显著地抑制细胞增殖率（Control *vs* siRNA at day 5, A549: 0.783±0.032 *vs* 0.568±0.028, *P* < 0.05; H460: 1.397±0.051 vs 1.076±0.056, *P* < 0.05）和集落形成能力（Control *vs* siRNA, A549: 426±37.3 *vs* 135±26.8, *P* < 0.05; H460: 257±35.5 *vs* 118±20.3, *P* < 0.05）（图 2A和图 2B）。为了验证LRRC3B对细胞侵袭的影响，使用A549和H460细胞进行基底膜基质侵袭实验。图 2C显示，与对照组相比，细胞转染LRRC3B后侵袭能力明显降低（A549: 59.7%; H460: 65.1%）。

**2:**
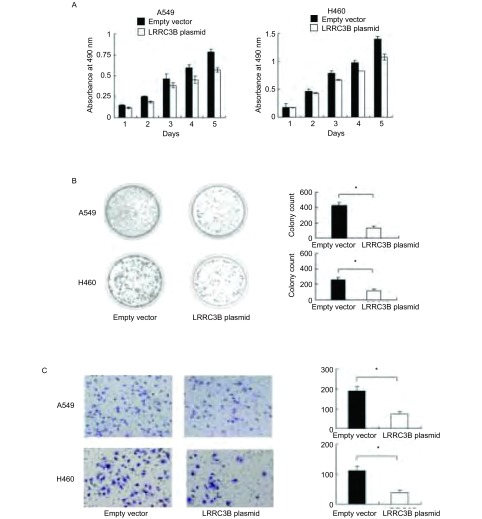
恢复LRRC3B表达抑制细胞增殖和侵袭。A：转染LRRC3B质粒的A549和H460细胞进行MTT实验；B：转染LRRC3B质粒以及空白对照的细胞进行集落形成实验，结果显示恢复LRRC3B表达明显降低集落形成能力；C：恶性侵袭实验显示A549和H460细胞中转染LRRC3B后降低细胞侵袭能力。^*^P < 0.05。 Upregulating the expression of LRRC3B inhibits cell growth and invasion. A: MTT assays of A549 and H460 cells transfected with LRRC3B; B: Colony formation assays of A549 and H460 cells transfected with LRRC3B; C: Transwell result shows that LRRC3B transfection weakens the activity of invasion of A549 and H460 cells. ^*^P < 0.05.

### 2.2 LRRC3B抑制细胞周期进程并调控cyclin D1和MMP9表达

之前实验结果显示LRRC3B导致细胞增殖和侵袭能力的降低。我们进一步分析LRRC3B在细胞周期进程中的角色，如图 3A所示，在H460和A549细胞系中LRRC3B上调能够抑制细胞G_1_期到S期的推进。为了了解可能的机制，我们检测了一系列与生长和侵袭相关的蛋白。如图 3B所示，转染LRRC3B明显抑制细胞周期蛋白cyclin D1以及与侵袭相关的蛋白MMP9的表达。

**3:**
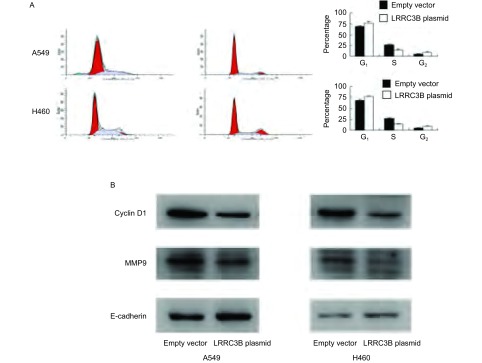
转染LRRC3B抑制细胞周期进程，下调cyclin D1和MMP9表达量。A：细胞周期分析显示转染LRRC3B降低S期细胞比率，增加G_1_期细胞比率；B：Western blot分析显示恢复LRRC3B的水平可以减少cyclin D1和MMP9蛋白的表达。 LRRC3B transfection inhibits the process of cell cyle and downregulates the expression of cyclin D1 & MMP9. A: The analysis of cell cycle reveals that LRRC3B transfection decreases the rate of S phase and increases the rate of G_1_ phase; B: The analysis of Western blot shows that LRRC3B downregulates the expression of cyclin D1 and MMP9.

### 2.3 LRRC3B抑制细胞的增殖和侵袭

在肺癌细胞系H3255中通过siRNA敲除LRRC3B，进一步分析LRRC3B的生物学功能。如图 4A-图 4D所示，LRRC3B敲除后提高了H3255的增殖率，提升了细胞的集落形成能力以及侵袭能力。另外，在LRRC3B敲除的细胞中，MMP9和cyclin D1的表达略微上调（图 4E）。

**4:**
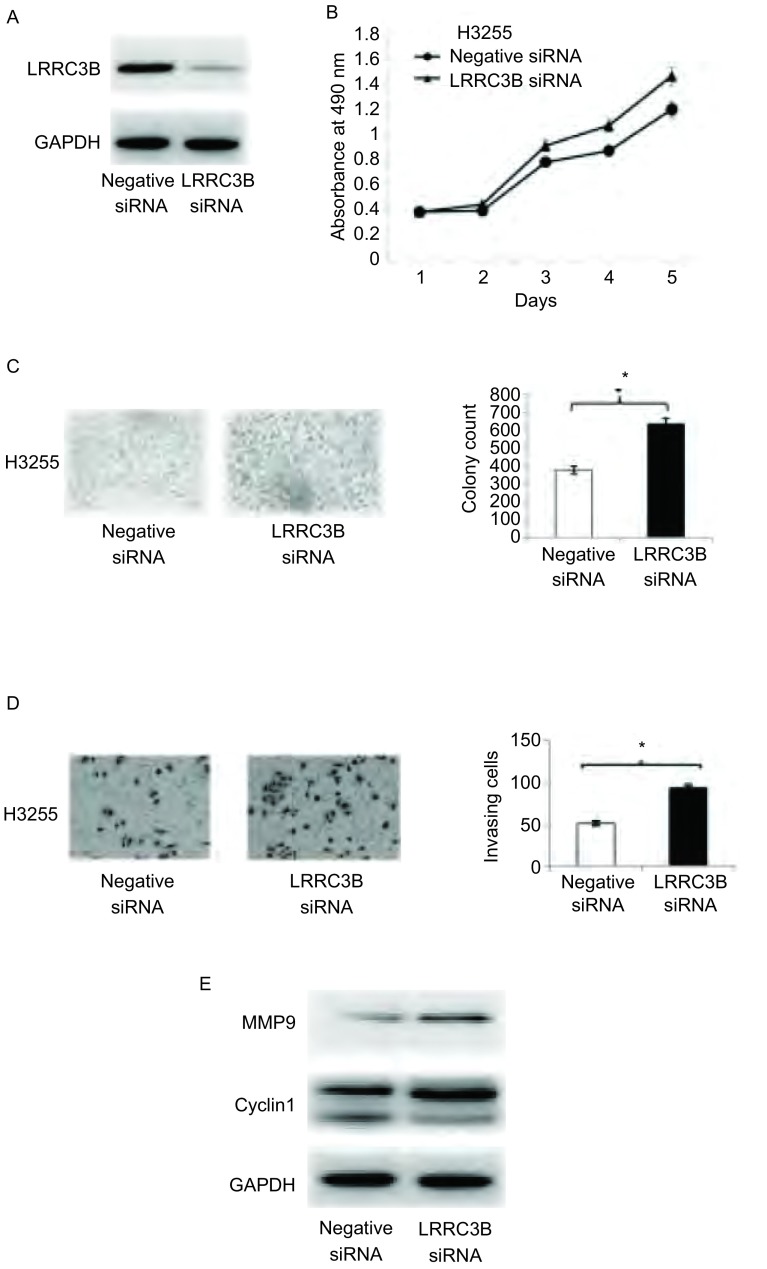
LRRC3B抑制细胞生长侵袭并下调cyclin D1和MMP9的表达。A：Western blot分析显示，与对照组相比，LRRC3B siRNA明显降低H3255细胞中LRRC3B的水平；B：MTT实验显示LRRC3B抑制细胞生长；C：集落形成实验显示LRRC3B减少细胞集落形成数目；D：侵袭实验显示LRRC3B抑制H3255细胞侵袭；E：Western blot分析显示，LRRC3B敲除略微上调cyclin D1和MMP9的表达。^*^P < 0.05。 LRRC3B inhibits cell growth & invasion and downregulates the expression of cyclin D1 & MMP9. A: The analysis of Western blot reveals that LRRC3B siRNA downregulates the expression of LRRC3B in H3255 cells; B: MTT assays shows that LRRC3B inhibits cell growth; C: Colony formation assays shows that LRRC3B reduces the quantity of cell colony; D: Transwell assays reveals that LRRC3B inhibits cell invasion; E: The analysis of Western blot shows that LRRC3B siRNA upregulates the expression of cyclin D1 and MMP9 tinily. ^*^P < 0.05.

## 3 讨论

通过对一些癌症患者的调查表明，LRRC3B失活是由甲基化或去甲基化导致的。在胃癌，结直肠癌和透明细胞肾细胞癌中发现LRRC3B mRNA表达降低且LRRC3B的启动子区域去甲基化。在胃癌细胞中通过转染上调LRRC3B的表达水平能够抑制集落形成^[12, 13]^。然而，截至目前还没有研究涉及LRRC3B蛋白在肺癌组织中的表达水平及其与临床病理参数的相关性。另外，LRRC3B在人类NSCLC中的生物学功能尚不明确。我们的研究显示与正常支气管细胞NHBE相比，9株肺癌细胞系中有4株细胞系其LRRC3B的表达水平偏低。通过LRRC3B质粒转染，在LRRC3B表达较低的NSCLC细胞株中恢复内源性表达，结果显示LRRC3B能够显著抑制细胞增殖和细胞周期进展。LRRC3B在肾细胞癌中也可以抑制集落的形成^[14]^。我们的结果与这些研究保持一致，提示LRRC3B在肺癌细胞中是一种重要的肿瘤抑制基因。至今为止，LRRC3B在细胞周期进程和细胞侵袭活动中的作用研究尚不透彻。LRRC3B作为一个肿瘤抑制基因的机制尚未阐明。因此，我们调查细胞周期进程及其相关蛋白并发现LRRC3B上调能够降低S期百分比和cyclinD1表达量。CyclinD1在细胞周期进程G_1_到S期的过程中发挥重要作用。许多研究^[15, 16]^证明cyclinD1在NSCLC中过表达并与恶性增殖和预后不良相关。这些结果表明LRRC3B可以通过调节cyclinD1的表达来调控细胞周期进程。LRRC3B在细胞侵袭过程中的作用之前还没有报导。在本次课题研究中，我们发现LRRC3B能够抑制肺癌细胞的侵袭能力。LRRC3B转染能够下调MMP9的表达，MMP9在许多类型的癌症中是入侵的一个重要中介^[17-20]^。因此，LRRC3B抑制细胞侵袭的功能可能是通过下调MMP9的表达来实现的。

综上所述，这项研究证明在NSCLC中LRRC3B蛋白水平下调，恢复肺癌细胞系中LRRC3B的表达可以抑制细胞增殖和侵袭，作用机制可能是通过调控cyclinD1和MMP9表达量来实现的。综合这些发现，我们推断LRRC3B是NSCLC中非常重要的肿瘤抑制基因，通过抑制细胞增殖以及侵袭的方式来调控肿瘤的发展进程。进一步研究其分子机制，分析他与临床病理参数的相关性，可能会为肿瘤治疗提供一个新的靶点。
